# Paprika-Induced Hypersensitivity Pneumonitis: A Report of a Rare Case

**DOI:** 10.7759/cureus.80195

**Published:** 2025-03-07

**Authors:** Xinyu Fu, Chunyu Liu, Pihua Gong, Zhancheng Gao

**Affiliations:** 1 Department of Pulmonary and Critical Care Medicine, Peking University People's Hospital, Beijing, CHN

**Keywords:** capsaicin, hypersensitivity pneumonitis, interstitial lung disease, paprika, treatment

## Abstract

Hypersensitivity pneumonitis (HP) is an interstitial lung disease caused by exposure to environmental antigens in susceptible individuals. It is a type IV hypersensitivity reaction. The antigens involved in HP are numerous, but their identification is challenging. We report the first clinical case of HP associated with exposure to paprika. The diagnosis was confirmed based on the patient's exposure history, chest imaging, bronchoalveolar lavage fluid analysis, and pathological findings. Avoidance of antigens and treatment with glucocorticoids resulted in symptom relief. Follow-up chest imaging showed no ideal improvement; immunosuppressants or antifibrotic drugs may be considered in combination if necessary. The case highlights that individuals working in paprika production and processing should take preventive measures to avoid chronic pulmonary fibrosis.

## Introduction

Hypersensitivity pneumonitis (HP) is an inflammatory disease affecting the lung parenchyma and small airways, caused by repeated inhalation of organic dust in susceptible individuals [1]. It is the third most common interstitial lung disease (ILD), following idiopathic pulmonary fibrosis (IPF) and connective tissue disease (CTD)-related ILD [2]. Now, HP is classified into non-fibrotic and fibrotic subtypes [1]. Non-fibrotic HP is characterized by ground-glass opacities and mosaic attenuation on imaging, with histopathological findings of bronchiolocentric chronic inflammation. In contrast, fibrotic HP exhibits patterns resembling usual interstitial pneumonia (UIP), fibrotic nonspecific interstitial pneumonia (NSIP), or other interstitial patterns.

Numerous antigens can provoke conditions such as farmer's lung, humidifier lung, and bird fancier's lung (BFL). However, the specific exposure factors remain unclear in some cases. BFL is the most common form, accounting for 66-68% of HP, with an incidence rate of 6-20% among individuals who handle pigeons [3]. Paprika, as a type of plant-based dust, has the potential to cause HP. As the primary bioactive component of paprika, capsaicin induces pulmonary inflammatory responses and small airway fibrosis, demonstrating respiratory toxicity in both animal models and humans [4]. Chronic exposure to capsaicin-containing aerosols may constitute the central mechanism underlying HP development.

This study is the first to fully report a clinical case of HP caused by paprika, presenting a comprehensive exposure-response evidence chain.

## Case presentation

A 55-year-old man presented to the hospital with a one-year history of chest tightness and wheezing. His symptoms were activity-related, with coughing and sputum production. He had been admitted one month earlier for the treatment of presumed chronic asthmatic bronchitis; he was prescribed ampicillin, aminophylline, and a seven-day course of 40 mg methylprednisolone.

The patient denied having any pre-existing medical conditions and had quit smoking for more than 30 years. He was involved in paprika processing in an enclosed poorly ventilated space. This resulted in 17 years of unprotected exposure to paprika dust. Before starting production, the chilies were properly stored to ensure no deterioration or mold growth. He denied exposure to animals, birds, humidifiers, or mold at home or in the workplace, as well as occupational histories such as carpentry, painting, coal-related, or farm work.

Upon assessment, his oxygen saturation was 96% on room air, his temperature was 36.7°C, his heart rate was 75 beats/min, and his blood pressure was 129/76 mmHg. On examination, fine inspiratory crackles were heard at the lung bases bilaterally, along with finger clubbing. There was no evidence of leg edema, oral ulcers, rashes, or joint swelling. Cardiovascular and abdominal examinations were unremarkable.

The patient showed normal leukocytes and elevated C-reactive protein and erythrocyte sedimentation rate. Test results for anti-double-stranded DNA antibody, extractable nuclear antigen panel, and rheumatoid factor were negative. Serum *Aspergillus* immunological IgG antibody test and *Aspergillus* galactomannan assay were negative (Table 1).

**Table 1:** Blood test results on admission CRP: C-reactive protein; ESR: erythrocyte sedimentation rate; LDH: lactate dehydrogenase; IgE: immunoglobulin E; ANA: anti-nuclear antibody; ENA: extractable nuclear antigen; RF: rheumatoid factor; IgG: immunoglobulin G

Test	Result	Normal value/unit
CRP	22.8	0-10 mg/L
ESR	38	0-15 mm/h
White blood cells	7.93	3.5-9.5x10^9^/L
Neutrophils	6.10	1.8-6.3x10^9^/L
Eosinophils	0.36	0.02-0.52x10^9^/L
D-dimer	60	0-243 ng/ml
Albumin	38.7	40-55 g/L
Creatinine	83	59-104 umol/L
LDH	295	109-245 U/L
Neuron-specific enolase	20	0-16.3 ng/ml
Cyfra 21-1	13.5	<3.3 ng/ml
IgE	2.57	<100 IU/ml
ANA	Negative	
ENA antibody	Negative	
RF	8.4	0-14 IU/ml
*Aspergillus* IgG antibody	Negative	
*Aspergillus* galactomannan assays	0.19	<0.5

**Figure 1:**
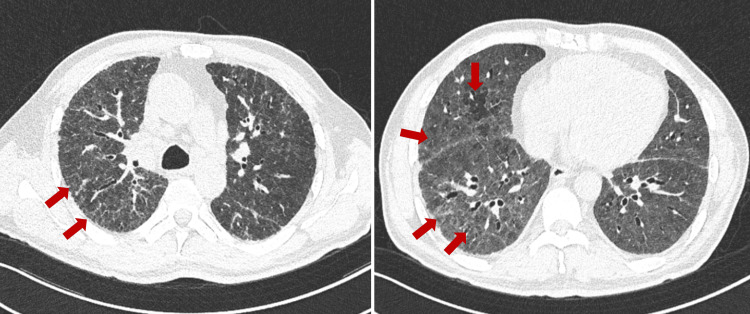
Chest CT on admission Pulmonary fibrosis (reticular opacities and traction bronchiectasis due to fibrosis formation) and headcheese sign (ground-glass opacities, normally ventilated lung tissue, and areas of air trapping). CT: computed tomography

Chest computed tomography (CT) revealed diffuse ground-glass opacities in both lungs, with interlobular septal thickening, mosaic attenuation, and traction bronchiectasis due to fibrosis formation (Figure 1).

Lung function tests indicated mild restrictive ventilatory impairment and diffusion dysfunction. The diffusing capacity for carbon monoxide (DLCO) is only 36.6% of the predicted value (Table 2).

**Table 2:** Pulmonary function test results FVC: forced vital capacity; FEV1: forced expiratory volume in one second; MEF: maximal expiratory flow; DLCO SB: single breath diffusing capacity of the lung for carbon monoxide; VA: alveoli volume

	Actual	Predicted value (Pred)	% Pred
FVC (L)	4.02	2.74	68.2
FEV1 (L)	3.23	2.49	77.2
FEV1 % FVC (%)	83.57	90.81	108.7
MEF 75/25 (L/s)	3.63	5.05	138.9
DLCO SB (mmol/min/kPa)	9.23	3.38	36.6
DLCO/VA (mmol/min/kPa/L)	1.42	0.86	60.6

**Figure 2:**
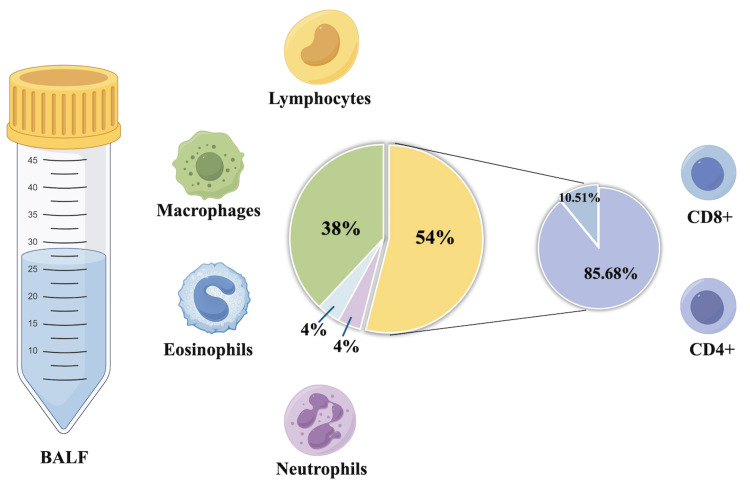
BALF cell classification and count The BALF analysis revealed a lymphocyte proportion of 54% (>40%) and macrophages at 38%. Flow cytometry identified CD4+ T cells comprising 85.56% and CD8+ T cells at 10.51%, with a CD4/CD8 ratio of 8.51. This figure was created by the authors using Figdraw (Home for Researchers, Hangzhou, Zhejiang Province, China). BALF: bronchoalveolar lavage fluid

The patient underwent a bronchoscopy examination. Bronchoalveolar lavage fluid (BALF) from the left lower lobe of the lung showed an elevated lymphocyte count of 54%. Lymphocyte subset analysis revealed an elevated CD4/CD8 ratio (Figure 2).

**Figure 3:**
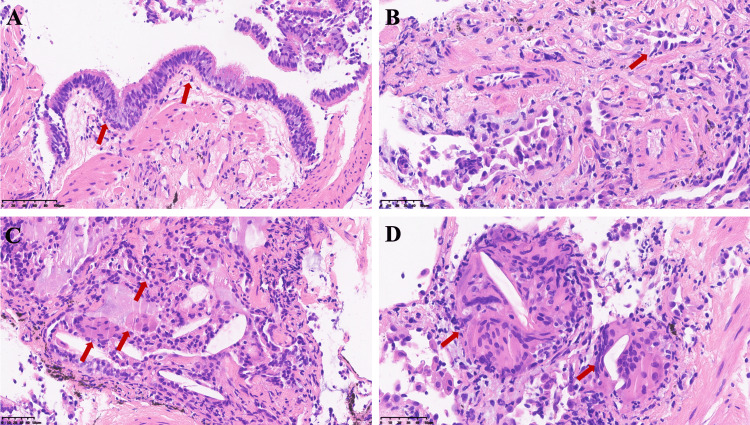
Histopathological findings from TBLC of the posterior basal segment of the right lower lobe: hematoxylin and eosin staining (A, B) Bronchial and alveolar epithelial cells show active proliferation. Interstitial space reveals chronic inflammatory cell infiltration and collagen deposition. (C, D) Fibrous tissue proliferation is evident, accompanied by granulomas, multinucleated giant cells, and cholesterol crystal deposits. TBLC: transbronchial lung cryobiopsy

BALF cultures yielded no significant pathogens. The next-generation sequencing of pathogen nucleic acids and qualitative DNA amplification tests for fungal pathogens in BALF were both negative. The pathological specimens from the transbronchial lung biopsy showed early chronic fibrotic interstitial pneumonia, with focal fibrosis, alveolar epithelial hyperplasia, occasional cholesterol crystals, and multinucleated giant cell reactions (Figure 3).

**Figure 4:**
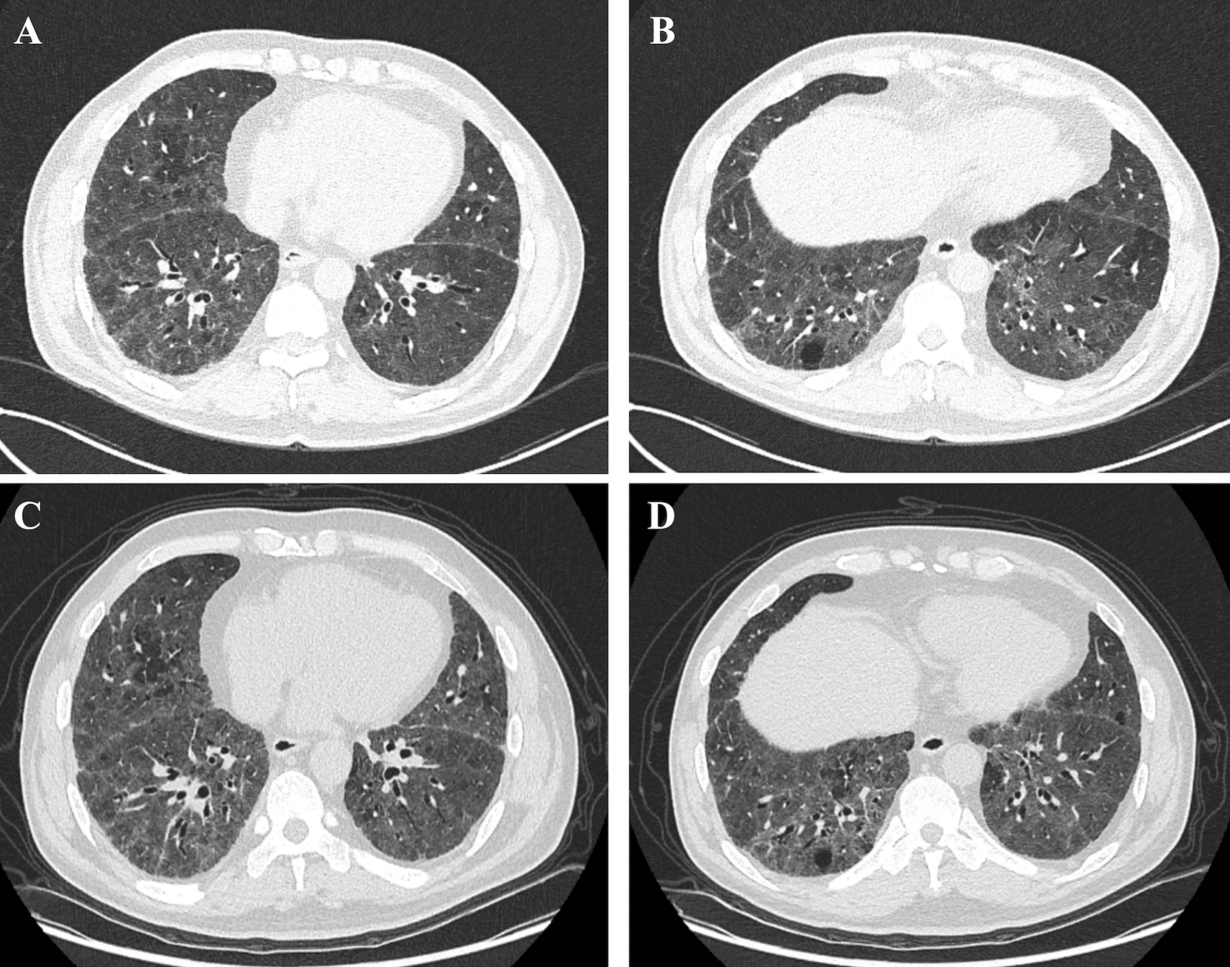
Post-treatment chest CT (A, B) Chest CT after avoiding exposure to chili powder for one month revealed a reduction in inflammation, although fibrosis persisted. (C, D) At the fifth-month follow-up, no significant changes in interstitial inflammation were observed in both lungs compared to the prior scan. CT: computed tomography

Based on symptoms and examination results, the diagnosis of paprika-induced HP is confirmed. The patient was advised to minimize exposure to paprika. Initial treatment included oral prednisone at a starting dose of 40 mg once daily with the dosage to be gradually tapered by 5 mg every two weeks. At the one-month follow-up, the patient's symptoms had significantly improved, with CT scans showing reduced inflammation but persistent interstitial fibrosis. By five months, pulmonary function tests indicated a slight improvement in ventilation with no significant change in gas exchange, and CT findings remained stable (Figure 4).

## Discussion

In this case, based on exposure assessment, lymphocytosis in BALF, imaging, and pathological findings, the diagnosis meets the criteria for HP. Chest CT revealed mosaic attenuation, air trapping, and diffuse axial distribution, while histopathology showed non-necrotizing granulomas and multinucleated giant cells. These are key features for distinguishing fibrotic HP from other ILDs [2]. Lymphocytosis greater than 30% in BALF is also a reasonable threshold for differentiating HP from IPF or sarcoidosis [5]. Negative serology, no extrapulmonary symptoms, and no relevant medication history help exclude CTD-associated ILD and drug-induced ILD.

Different exposure antigens may lead to varying prognoses for the disease [6], so identifying the antigens is a crucial step in the diagnostic process. Common methods include the following: (1) detailed exposure history which can be obtained using the CHEST Interstitial and Diffuse Lung Disease Patient Questionnaire [7]; (2) serum-HP antigen panel testing which detects IgG in the patient's serum against up to eight antigens (or more extensive panels that include 12 antigens, predominantly avian proteins); and (3) environmental antigen screening which has limitations in that positive results may reflect exposure rather than causality [8]. The only positive clue in the patient's questionnaire was a 17-year history of paprika production. A previous study suggested that "paprika splitter's lung" may be related to mold from improperly stored chili peppers [9]. *Aspergillus* IgG antibody tests, galactomannan assays, and DNA amplification tests were all negative in our case, ruling out the possibility of mold as the causative agent. Due to laboratory constraints, the serum-HP antigen panel was not performed, which constitutes a limitation of our study.

Paprika was identified as a plausible antigen, with the bioactive component capsaicin playing a key role. Chronic exposure to capsaicin can induce oxidative stress, generating reactive oxygen species that damage cells and intensify the inflammatory response in the respiratory tract [10]. Furthermore, increased sensitivity to capsaicin has been identified as a risk factor for severe asthma. Considering capsaicin as a potential trigger for HP, we measured its concentration in BALF. Regrettably, when BALF was sent for analysis, the capsaicin levels were below the detection limit. Due to the patient's prolonged absence from the antigen and the short half-life of capsaicin, it was likely metabolized and became undetectable [11].

BALF analysis is an important tool for evaluating and distinguishing HP. In a recent Delphi study, lymphocytosis greater than 40% in BALF is considered "important" or "very important" for the diagnosis of chronic HP [12]. Some studies suggested a more modest increase in lymphocytes in chronic HP, with levels potentially even normal. Lymphocyte counts may reflect different histopathological types, and higher lymphocyte counts are typically observed in organizing pneumonia and NSIP, while moderate increases are seen in UIP [13]. Additionally, the T-lymphocyte subtype in the patient's BALF shows an increased CD4/CD8 ratio. Chronic HP shows a shift from Th1 to Th2 microenvironment, which correlates with pulmonary fibrosis and appears to characterize the later stages of the disease [14]. T cells from chronic HP patients, particularly CD8+ T cells, exhibit clear signs of exhaustion and a reduction in cytotoxic activity.

## Conclusions

In our case, we conducted a thorough assessment of potential antigens. BALF showed a high proportion of lymphocytes, and imaging findings revealed the typical "headcheese sign" and traction bronchiectasis due to fibrosis formation. Early chronic fibrotic interstitial pneumonia and multinucleated giant cell reactions were observed in pathological examination. Based on these, the diagnosis of fibrotic HP caused by paprika was confirmed. Capsaicin, as an active biological component, played an important role. Early diagnosis and prompt avoidance of antigen exposure are crucial for the patient's prognosis. Monitoring disease progression and early identification of fibrosis risk are of paramount importance. Factors such as antigen avoidance, immune response, and individual susceptibility may influence the prognosis and progression of fibrosis. If fibrosis progresses, treatment with immunosuppressants and antifibrotic agents may be considered.
